# Applications of Signal Processing Techniques to Bioinformatics, Genomics, and Proteomics

**DOI:** 10.1155/2009/250306

**Published:** 2009-03-16

**Authors:** Erchin Serpedin, Javier Garcia-Frias, Yufei Huang, Ulisses Braga-Neto

**Affiliations:** 1 Department of Electrical and Computer Engineering, Texas A&M University, College Station, TX 77843-3128, USA; 2 Department of Electrical and Computer Engineering, University of Delaware, Newark, DE 19716-7501, USA; 3 Department of Electrical and Computer Engineering, University of Texas at San-Antonio, TX 78249-2057, USA

## 

The recent development of high-throughput molecular genetics technologies has brought a major impact to bioinformatics and systems biology. These technologies have made possible the measurement of the expression profiles of genes and proteins in a highly parallel and integrated fashion. The examination of the huge amounts of genomic and proteomic data holds the promise for understanding the complex interactions between genes and proteins, the functional processes of a cell, and the impact of various factors on a cell, and ultimately, for enabling the design of new technologies for intelligent management of diseases.

This special issue focuses on modeling and processing of data arising in bioinformatics, genomics, and proteomics using signal processing methods. The importance of signal processing techniques is due to their important role in extracting, processing, and interpreting the information contained in genomic and proteomic data. It is our hope that signal processing methods will lead to new advances and insights in uncovering the structure, functioning and evolution of biological systems.

The special issue consists of nine papers that span a wide range of problems and applications in bioinformatics, genomics, and proteomics such as design of compressive sensing microarrays, analysis of missing values in microarray data, and effect of imputation techniques on post genomic inference methods, RNA sequence alignment, detection of periodicity in genomic sequences and gene expression profiles, clustering and classification of gene and protein expression data, and intervention in probabilistic Boolean networks. Next, we will briefly introduce the papers reported in this special issue.

W. Dai et al. analyze how to design a microarray that it is fit for compressive sensing and that captures also the biochemistry of probe-target DNA hybridization. Algorithms and design results are reported for determining probe sequences that satisfy the binding requirements and for evaluating the target concentrations.

M. S. B. Sehgal et al. address the general problem of improving post genomic knowledge discovery procedures such as the selection of the most significant genes and inference of gene regulatory networks using missing microarray data imputation techniques. It is shown that instead of neglecting missing data, recycling microarray data via robust imputation techniques can yield substantial performance improvements in the subsequent post genomic discovery procedures.

B.-J. Yoon developed a novel efficient and robust approach for fast and accurate structural alignment of RNAs, including pseudoknots. The proposed method turns out to accelerate the dynamic programming algorithm for family-specific models such as profile-csHMMs and CMs, and to be robust to small parameter changes that are present in the model used to predict the constraint.

The paper by J. Epps explains in detail the origins of ambiguity in period estimation for symbolic sequences, and proposes a novel hybrid autocorrelation-IPDFT technique for periodicity characterization of sequences.

W. Zhao et al. developed a novel algorithm for identification of genes involved in cyclic processes by combining gene expression analysis and prior knowledge. The proposed cyclic-genes detection algorithm is validated on data sets corresponding to *Saccharomyces cerevisiae* and *Drosophila melanogaster*, and shown to represent a valuable technique for unveiling pathways related to cyclic processes.

T. J. Hestilow and Y. Huang propose a novel method for gene clustering using the shape information of gene expression profiles. The shape information which is represented in terms of normalized and time-scaled forward first-order differences is then exploited by a variational Bayes clustering approach and a non-Bayesian (Silhouette) cluster statistic, and shown to yield promising results in clustering time-series microarray data.

The paper by W. Zhao et al. proposes a new clustering approach to combine the traditional clustering methods with power spectral analysis of time series gene expression measurements. Simulation results confirm that the proposed clustering approach provides superior performance relative to hierarchical, K-means, and self-organizing maps, and yields additional information about temporal regulated genetic processes, for example, cell-cycle.

T. T. Vu and U. M. Braga-Neto address the important problem of assessing the effectiveness of bagging in the classification of small-sample genomic and proteomic data sets. Representative experimental results are presented and discussed.

Finally, the paper by B. Faryabi et al. studies the effects on intervention performance in the context of probabilistic Boolean networks due to a reduction in the values of the model parameters.

